# Correction to: LncMAPK6 drives MAPK6 expression and liver TIC self-renewal

**DOI:** 10.1186/s13046-019-1501-8

**Published:** 2019-12-20

**Authors:** Guanqun Huang, Hui Jiang, Yueming He, Ye Lin, Wuzheng Xia, Yuanwei Luo, Min Liang, Boyun Shi, Xinke Zhou, Zhixiang Jian

**Affiliations:** 1 Department of General Surgery, The Fifth Affiliated Hospital of Guangzhou Medical University, Guangdong Sheng, China; 2 Department of Abdominal Oncology, The Fifth Affiliated Hospital of Guangzhou Medical University, Guangdong Sheng, China; 3 Department of General Surgery, Guangdong General Hospital, Guangdong Academy of Medical Sciences, Guangdong Sheng, China

**Correction to: J Exp Clin Cancer Res**

**https://doi.org/10.1186/s13046-018-0770-y**

In the original publication of this article [1], the author found an error in Fig. 2f. lncGPR107 should be changed to lncMAPK6, and the corrected Fig. 2 is shown below.

**Fig. 2:**
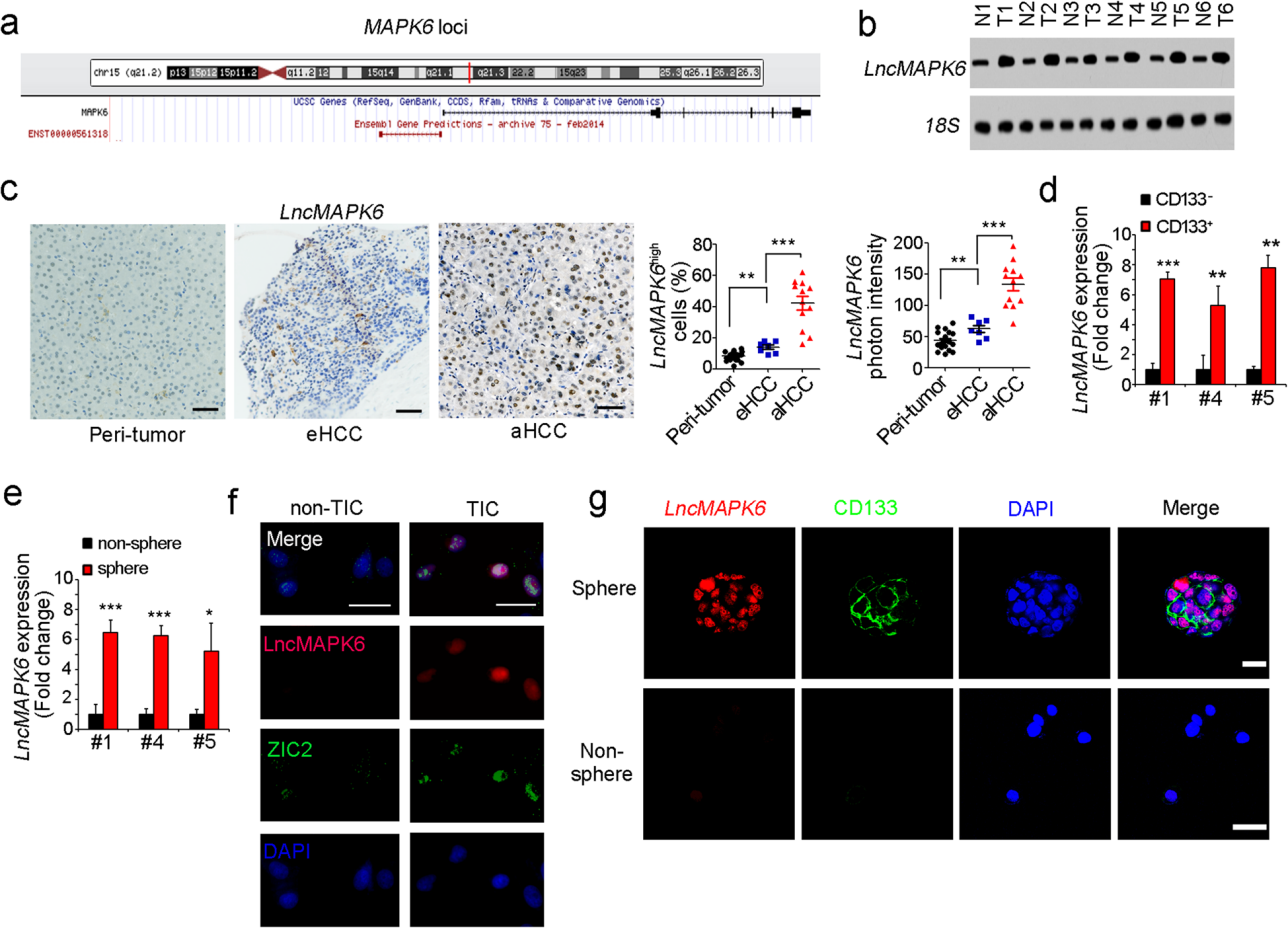
LncMAPK6 is overexpressed in liver tumor and TICs. **a** MAPK6 locus was shown. There is a divergent lncRNA (ENST00000561318, termed as lncMAPK6 in this research) in near from MAPK6 locus according to UCSC Genome Browser. **b** LncMAPK6 expression levels were detected using Northern blot. 18S rRNA was a loading control. **c** 19 peri-tumor, 7 eHCC and 12 aHCC were used for In situ hybridization (ISH). Representative photos and indicated ratios were shown. **d** LncMAPK6 expression levels in CD133^+^ liver TICs and CD133^−^ non-TICs were detected by realtime PCR, and non-TIC expression was used for data normalization. **e** Spheres and non-spheres were selected, and lncMAPK6 expression was examined with realtime PCR. **f**, **g** FISH assay confirmed the high expression of lncMAPK6 in liver TICs (**f**) and oncospheres (**g**). Zic2 and CD133 were markers of liver TICs. Scale bars, C, 50 μm; F, 20 μm

The author sincerely apologizes for the inconvenience caused to the readers.
